# The role of free eye health resources in the ongoing learning and development of eye health workers in Eastern Africa

**Published:** 2024-02-09

**Authors:** Elliott H Taylor, Tara Mtuy, Justus Rwiza, Sarity Dodson, Elmien Wolvaardt

**Affiliations:** 1 International Centre for Eye Health, London School of Hygiene & Tropical Medicine, London, UK.; 2 Assistant Professor: International Centre for Eye Health, London School of Hygiene & Tropical Medicine, London, UK.; 3 Senior Resident in Ophthalmology, KCMC Eye Department, Kilimanjaro Christian Medical University College, Moshi, United Republic of Tanzania; 4 Director of Research & Evidence: The Fred Hollows Foundation, Melbourne, Australia.; 5 Editor-in-Chief: Community Eye Health Journal, International Centre for Eye Health, London School of Hygiene and Tropical Medicine, UK.

**The *Community Eye Health Journal* has a broad readership in Eastern Africa and – along with other providers of free resources – makes a significant contribution to the ongoing learning and development of the eye health workforce.**

**Figure:**
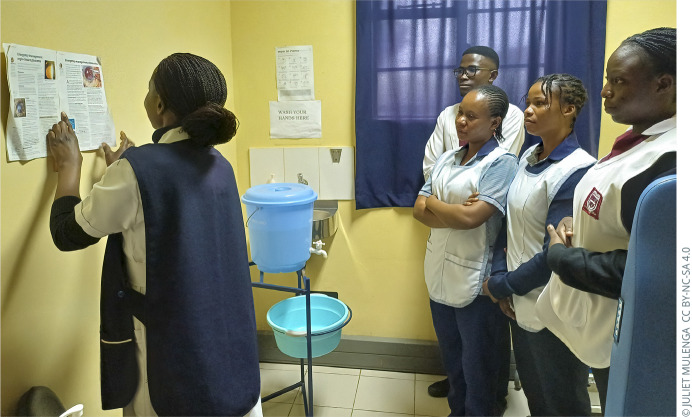
The *Community Eye Health Journal* is used to teach students nurses about different eye conditions and their management. zambia

Eastern Africa has a high prevalence of vision impairment and blindness, and a low number of ophthalmologists and optometrists per million population, compared to other global regions.^1^

The *Community Eye Health Journal* is available free of charge in print, online, and via a smartphone app. The journal, which is published by International Centre for Eye Health (ICEH) at the London School of Hygiene & Tropical Medicine, aims to contribute to the ongoing learning and development of eye health personnel in low-resource settings by producing relevant and up-to-date clinical and public health educational articles. The *Community Eye Health Journal* English and French editions together have more than 15,000 print subscribers, over 33,000 online users, and close to 800 smartphone app users based in Africa (Table 1).

**Table 1:** *Community Eye Health Journal* distribution globally, in Africa, and in East Africa (English and French editions).

CEHJ formats	Global	Africa	East Africa
**CEHJ print issue** (copies distributed)	17,153	15,112 (88.1%)	4,609 (26.9%)
**CEHJ website** (individual users)	238,877	33,693 (14.1%)	8,434 (3.5%)
**CEHJ app** (individual users)	1,536	796 (51.8%)	240 (15.6%)

Over the last year, eye health institutions in Kenya, Uganda, Ethiopia, Tanzania, and Rwanda, the College of Ophthalmologists of Eastern, Central and Southern Africa (COECSA), and the International Centre for Eye Health (ICEH) collaborated with The Fred Hollows Foundation to conduct a survey assessing training programs and the current eye health workforce in Eastern Africa. The survey forms part of a wider project to aid the development of a long-term regional strategy for the development of human resources for eye health in Eastern Africa.

The *Community Eye Health Journal* distributes 26.9% of its print copies to countries in East Africa (Table 1), but website and smartphone app users make up just 3.5% and 15.6%, respectively. As part of this survey, we asked respondents to complete an optional set of questions which aimed to assess how providers of free educational resources, including the print, online and smartphone app versions of the *Community Eye Health Journal*, were contributing to the ongoing learning and development of the eye health workforce in Eastern Africa. The four providers were:

- The *Community Eye Health Journal* (distributed as printed copies, online articles, and via a smartphone app): www.cehjournal.org
- Orbis Cybersight (asynchronous online courses and live webinars): www.cybersight.org
- ICEH Online Learning Courses (asynchrononous online courses): https://iceh.lshtm.ac.uk/oer-courses/
- The International Agency for the Prevention of Blindness (IAPB) online resources: www.iapb.org/learn/resources

## Results

Of the 375 eye care professionals working in Kenya, Uganda, Tanzania, Rwanda, and Ethiopia who submitted responses, 262 completed the optional CPD questions. Of these, 42.0% were optometrists or other refractive error workers, 29.0% were ophthalmologists, and 19.5% were nurses or allied eye care personnel.

Nearly four out of every five respondents (78.9%)belonged to a professional body, and 60.9% overall reported being required by that body to provide evidence of their ongoing learning (53.3% of the optometrists or other refractive error workers, 59.2% of the ophthalmologists, and 76.09% of the nurses and allied eye care personnel who responded).

### Access

Most respondents (72.8%) had reliable and affordable access to online resources, and smartphones were the most common way of accessing learning materials (82.4%). However, most of the respondents said they read *Community Eye Health Journal* articles in the print copy (52.3%), followed by the website (42.3%), and the smartphone app (22.5%). This could be a matter of preference, or because respondents were not familiar with the other formats. Indeed, only 46.2% of respondents were aware that the *Community Eye Health Journal* has a smartphone app. Many people accessed the journal both in print and electronically, reflecting qualitative findings from our 2015 survey: that people enjoyed the convenience of reading articles in a print version, but enjoyed the ease with which they could share the online articles with colleagues and students.^2^

### Ongoing learning and development

Respondents were asked which of the online and print resources they used for their ongoing learning and development. The results were:

- The *Community Eye Health Journal* (all formats): 61.5%
- Orbis Cybersight: 53.0%
- ICEH Open Online Courses: 27.9%
- The International Agency for the Prevention of Blindness (IABP) online resources: 26.0%.

However, fewer respondents reported using these resources to provide **evidence of their learning** to their respective professional bodies:

- The *Community Eye Health Journal* (all formats): 6.5%
- Orbis Cybersight: 5.3%
- ICEH Open Online Courses: 6.5%
- IAPB online resources: 3.1%.

## Discussion

This study shows that the *Community Eye Health Journal* is a major component of the ecosystem of providers of free resources for ongoing learning and development for the eye health workforce in Eastern Africa, alongside Orbis Cybersight, ICEH Open Online Courses, and IAPB's online resources.

**Figure:**
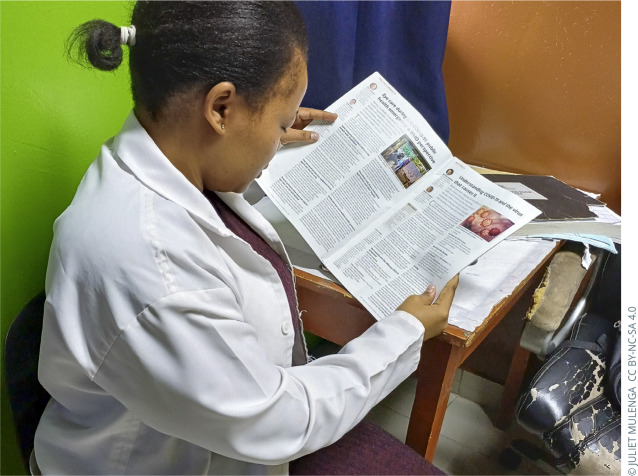
A second-year resident reads the *Community Eye Health Journal* in preparation for a presentation. zambia

### Evidence of ongoing learning

It is encouraging that more than half of the respondents engaged with the *Community Eye Health Journal* (61.5%) and/or Orbis Cybersight (53.0%) for their ongoing learning and development, and more than a quarter with ICEH's Open Online Courses and/or IAPB's online resources. However, only a small fraction of respondents reported using these to provide evidence of learning to their respective professional bodies. This may be due to current CPD evidence requirements: for example, in Tanzania, the respective professional councils for ophthalmologists, ophthalmic nurses, and optometrists do not currently recognise reading a relevant journal as evidence of ongoing learning, which may be attributed to the inherent challenges in verifying this type of activity. However, writing and publishing an article can be used as CPD evidence in Tanzania. For the *Community Eye Health Journal*, this is further confirmation of the value of encouraging and supporting eye care practitioners of all disciplines to contribute to the journal.

There is room to further improve eye care workers’ awareness of these free resources. Of the respondents who completed the additional questions, 17.2% had not yet heard about the *Community Eye Health Journal*. This figure was 25.2% for Cybersight, 51.9% for ICEH's Open Online Courses, and 48.9% for IAPB's online resources. There may be opportunities for these four providers to collaborate and cross-promote each other's resources.

**Figure:**
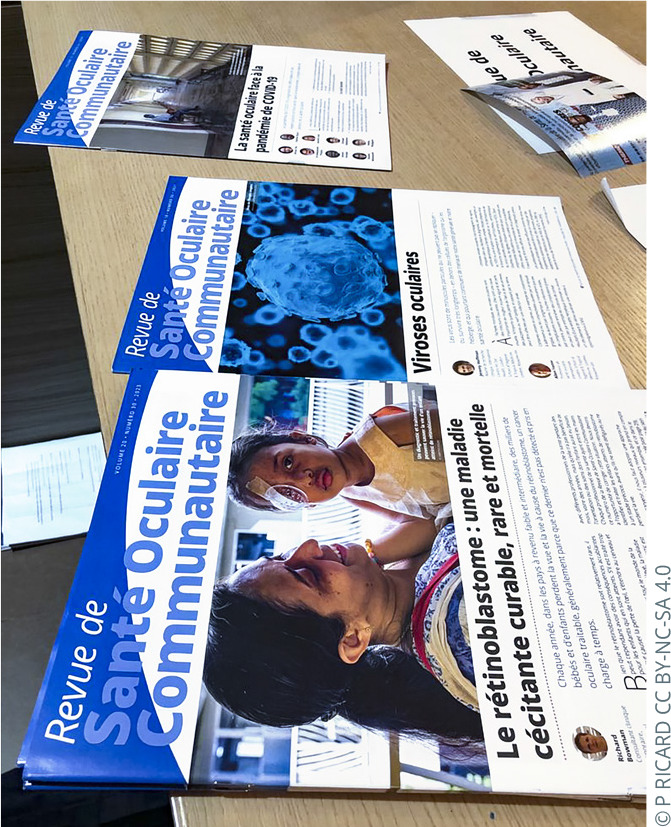
The French edition of the *Community Eye Health Journal* is sent to 475 readers in Eastern Africa.

Another opportunity for collaboration could centre around working with national professional bodies to develop innovative ways that eye care professionals’ engagement with these free resources can be submitted as evidence of their ongoing learning and professional development.

## Acknowledgements

The authors would like to thank the University of Nairobi (Kenya), Mbarara University of Science and Technology (Uganda), Bahir Dar University and Eyu-Ethiopia (Ethiopia), Kilimanjaro Christina Medical Centre (Tanzania), Rwanda International Institute of Ophthalmology (Rwanda), the College of Ophthalmologists of Eastern Central and Southern Africa (COECSA), The International Centre for Eye Health, The Fred Hollows Foundation.
